# Safety and efficacy of ultrasound-guided percutaneous A1 pulley release using a needle knife: An anatomical study

**DOI:** 10.3389/fsurg.2022.967400

**Published:** 2022-09-20

**Authors:** Zuyun Qiu, Hui Li, Yifeng Shen, Yan Jia, Xiaojie Sun, Qiaoyin Zhou, Shiliang Li, Weiguang Zhang

**Affiliations:** ^1^ Department of Traditional Chinese Medicine Bone-Setting, Beijing Jishuitan Hospital, Beijing, China; ^2^ Department of Acupuncture and Moxibustion, China-Japan Friendship Hospital, Beijing, China; ^3^ Department of Traditional Chinese Surgery, Hospital of Chengdu University of Traditional Chinese Medicine, Chengdu, China; ^4^ Chinese Medicine College, Fujian University of Traditional Chinese Medicine, Fuzhou, China; ^5^ Department of Human Anatomy, School of Basic Medical Sciences, Peking University, Beijing, China

**Keywords:** stenosing flexor tenosynovitis, needle-knife, anatomy, percutaneous release, ultrasound-guided technique, a1 pulley, trigger finger

## Abstract

**Objective:**

The present study aimed to assess the efﬁcacy and safety of ultrasound-guided percutaneous A1 pulley release using a needle knife.

**Methods:**

The author performed percutaneous A1 pulley release in 84 cadaveric hands fixed with 10% formalin. The cadaveric hands were divided into three groups: 28 hands in each group (group U: ultrasound-guided needle knife pushing group, group N: non-ultrasound-guided needle knife pushing group, group T: classical needle knife operation puncture group). Percutaneous A1 pulley release was performed, the soft tissue was dissected layer by layer, and the relevant anatomical data were measured.

**Results:**

The injured cases were as follows: group U, 29 (20.7%); group N, 36 (25.7%); and group T, 28 (20.0%). There is no significant difference between different tissue injury types in different intervention methods. The missed release cases were as follows: group U, 8 (5.7%); group N, 4 (2.9%); and group T, 13 (9.3%). The percentage of released A1 pulley were as follows: group U, 71.4% ± 30.7%; group N, 66.0% ± 20.3%; and group T, 61.0% ± 30.4%. The percentage of released A1 pulley of the three groups were compared: group U > group N > group T, and there was statistical difference between the three groups. The full release rates of the three groups were compared: group U(31.4%) > group N(15.7%) > group T(13.6%), and there were significant difference in the full release of A1 pulley between group U and group T, group N.

**Conclusion:**

Based on the cadaver specimen, the length and percentage of released A1 pulley is longer by ultrasound-guided percutaneous A1 pulley release using a needle-knife. and there was no statistical difference in the injury rate between the three techniques.

**Type of Study and Clinical Relevance:**

Clinical anatomic study. To test the efﬁcacy and safety of ultrasound-guided percutaneous A1 pulley release using a needle knife in cadaveric hands, and provide an anatomically based support in clinic.

## 1. Introduction

Stenosing flexor tenosynovitis, or trigger finger (TF), is one of the most common conditions seen in hand surgery practice. TF is an aseptic inﬂammatory process that involves the flexor digital tendon at the A1 pulley. The flexor digital tendon glides in a ﬁbro-osseous tunnel between the metacarpal, phalanges, and pulley. Inﬂammation or swelling of this tunnel can occur because of repetitive use, thus preventing smooth gliding of the tendon under the A1 pulley.

**Figure 1:**
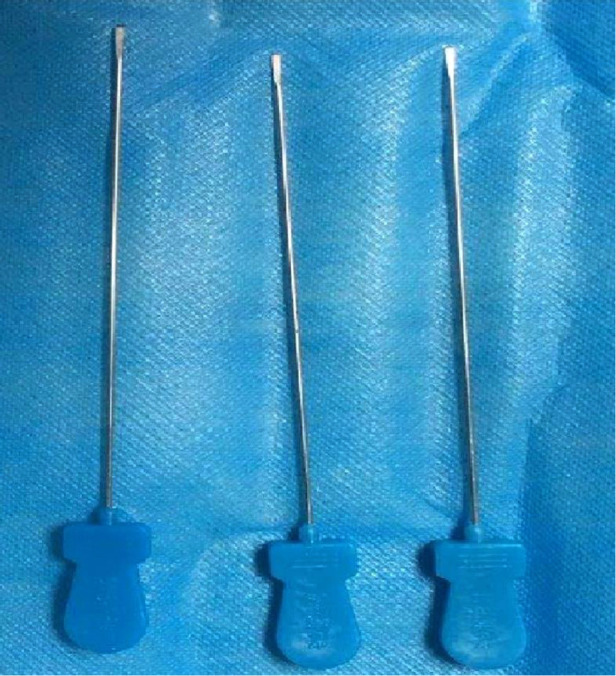
Needle-knife.

The most common conservative treatment is an intrathecal corticosteroid injection which provides satisfactory results in the early stages of TF. However, most patients do not accept treatment until they have been ill for a long time, so overall results with nonoperative management have been variable and disappointing (1–3). Percutaneous A1 pulley release was first reported by Lorthioir (4) in 1958. Percutaneous release avoids the time and expense of an outpatient surgical procedure and reduces the incidence of scar tenderness and possible infection. When conservative treatment fails to relieve triggering of the flexor tendons, percutaneous A1 pulley release is recommended as the first choice (5–7). There are many medical tools for percutaneous release, and the needle knife (Figure 1) which made in China is one of them. It is a miniature surgical instrument comprising a handle, needle body, and blade (8). It has been widely used clinically by doctors in China with satisfactory efficacy (9–11). The perceived disadvantages of such percutaneous release techniques include lack of direct visualisation, inability to ascertain complete release, and potential injury to important structures, including the digital vessels, nerves, and tendons. Furthermore, it is not conducive to learning and transmission (7–11). The visualisation of percutaneous release is important for future developments (12). Ultrasound-guided techniques are of great value in clinical practice (13, 14). At present, there are reports on the clinical study of ultrasound-guided percutaneous A1 pulley release (15); however, there are no reports on its anatomical research, which is not conducive to the clinical promotion of this technology.

The present study aimed to clarify the anatomical basis of ultrasound-guided percutaneous A1 pulley release using a needle knife and assess the efﬁcacy and safety of ultrasound-guided percutaneous A1 pulley release by needle knife in 84 cadaveric hands fixed with 10% formalin.

## 2. Materials and methods

### 2.1. Materials

Percutaneous A1 pulley release was performed in 84 cadaveric hands fixed with 10% formalin from November 2018 to May 2019. Of the 84 cadaveric hands, 54 belonged to males and 30 belonged to females, with a mean age of 82.6 ± 12.5 years (range, 24–101 years). All 420 ﬁngers (84 hands) were examined and confirmed to be intact without injury, operation history, and deformity. Appropriate institutional approval was obtained prior to this study. Then, the 84 hands (420 ﬁngers) were divided into three groups, with 28 hands (140 ﬁngers) in each group.

### 2.2. Methods

#### 2.2.1. Group

##### 2.2.1.1. Group U: ultrasound-guided needle knife pushing group

**Figure 2:**
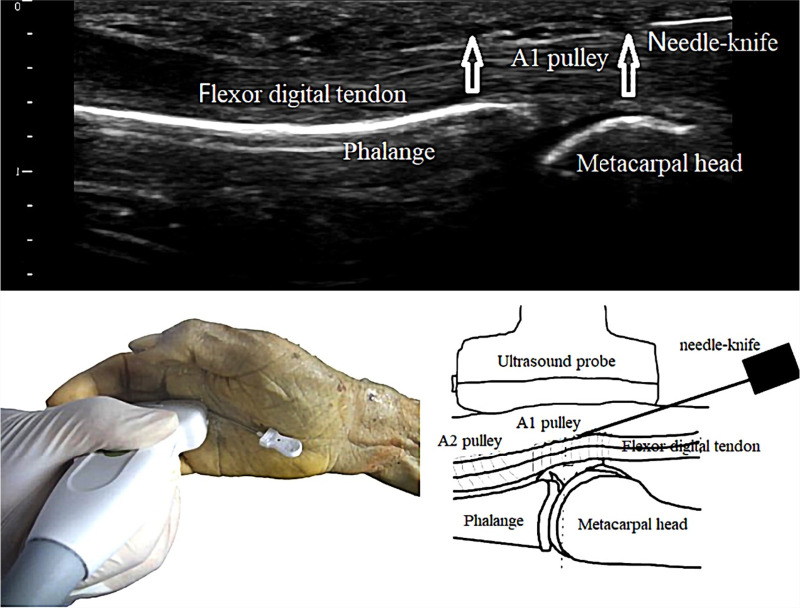
ultrasound-guided percutaneous A1 pulley release by needle-knife.

First, we placed the high-frequency ultrasound probe (Model: Wisonic-Navi, frequency:12 MHz, Supported by Shenzhen Wisonic Medical Technology Co., Ltd) longitudinally over the palmar striation at the metacarpophalangeal (MP) joint, followed by an ultrasound survey scan to identify the metacarpal bone, proximal phalanx, flexor tendon, and A1 pulley. Then, the needle knife was inserted 2 mm proximal to the transducer, at an angle of 15°, and the knife edge was oriented in the longitudinal axis of the tendon. The needle knife was then moved successively 8 mm in a proximal-to-distal manner to complete the release of the A1 pulley (Figure 2). All operations were performed under real-time ultrasound guidance. Completion of the release was assessed using ultrasound guidance.

##### 2.2.1.2. Group N: non-ultrasound-guided needle knife pushing group

First, we determined the appropriate site of entry for the needle knife by measuring and marking anatomical landmarks for the A1 pulley. The surface landmarks were described by Froimson (16) and Fiorini et al. (17). Froimson recommended starting at the MP crease of the thumb. Fiorini recommended utilising the proximal palmar crease as the starting point of the index finger, halfway between the proximal and distal palmar creases of the middle finger, and the distal palmar crease of the ring and little fingers. Then, the needle knife was inserted in a manner similar to that in group U. After puncturing the skin, the needle knife was advanced until a crunch was felt as the blade encountered the A1 pulley, following which the needle knife maintained at a constant depth in the tissues, and moved successively 8 mm in a proximal-to-distal manner to complete the release of the A1 pulley. Completion of the release was assessed by the loss of the grating sensation.

##### 2.2.1.3. Group T: classical needle knife operation puncture group

**Figure 3:**
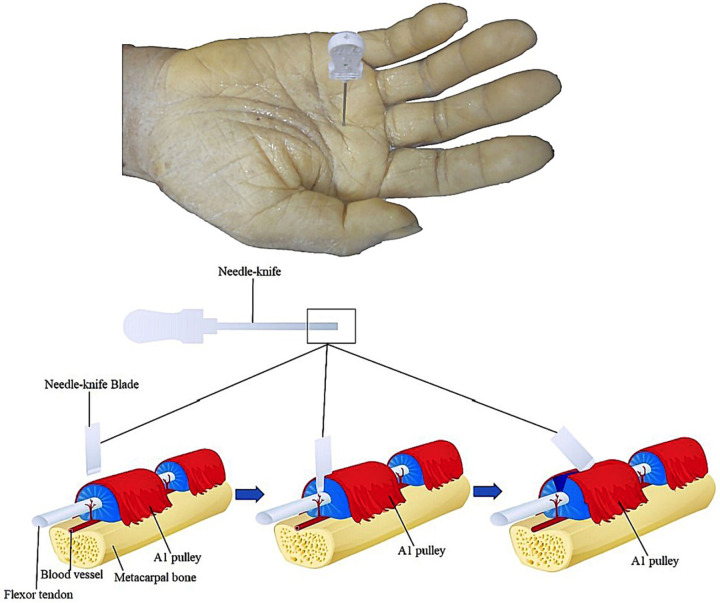
Schematic diagram of classical needle knife operation.

The appropriate site of entry for the needle knife was the same as that in group N. The needle knife was inserted at an angle of 90°, and the knife edge was oriented in the longitudinal axis of the tendon. After puncturing the skin, the needle knife was advanced until the loss of the grating sensation. The needle knife was then withdrawn and moved slightly in a proximal-to-distal manner, and the previous steps were repeated to release the A1 pulley. Completion of the release was assessed by the loss of the grating sensation (Figure 3).

#### 2.2.2. Types of outcome measures

After the release of the A1 pulley, a longitudinal incision was made, extending from the proximal interphalangeal joint to the midpalmar crease in line with the respective rays, and dissection was carefully performed down to the tendon sheath. Then, each researcher assessed the injury of the digital neurovascular bundles, A2 and palmar aponeurosis (PA) pulley, and flexor digital tendons and measured the length of the released A1 pulley and the actual lengths of the A1 pulleys. Measurements were completed using Vernier digital callipers (Pittsburgh by Harbor Freight Tools, Camarillo, CA) with a measurement error of 0.01 mm.

##### 2.2.2.1. Safety

**Figure 4:**
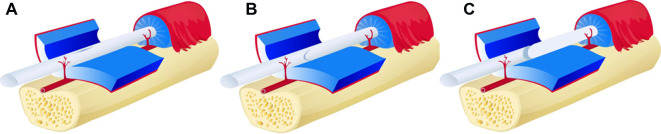
(**A**) longitudinal tendon scoring (indentation into the tendon substance), (**B**) partial laceration (interruption of one edge of the tendon with the tendon continuity maintained), (**C**) complete laceration (interrupted tendon continuity).

The safety index refers to the injury condition outside the target (A1 pulley). First, the digital neurovascular bundles and A2 and PA pulley were assessed as normal (if no cut was made) or injured (if the needle knife had made a cut). The flexor digital tendons were inspected to evaluate any injury. Tendon injuries (Figure 4) were classiﬁed as no injury, longitudinal tendon scoring (indentation into the tendon substance), partial laceration (interruption of one edge of the tendon with the tendon continuity maintained), or complete laceration (interrupted tendon continuity).

The number of digital neurovascular bundles, A2 and PA pulley, flexor digital tendons, and the total number of injuries in each group were counted. The injury rate was calculated as injury rate (%) = number of injuries ÷ total cases × 100%.

##### 2.2.2.2. Efficacy

**Figure 5:**
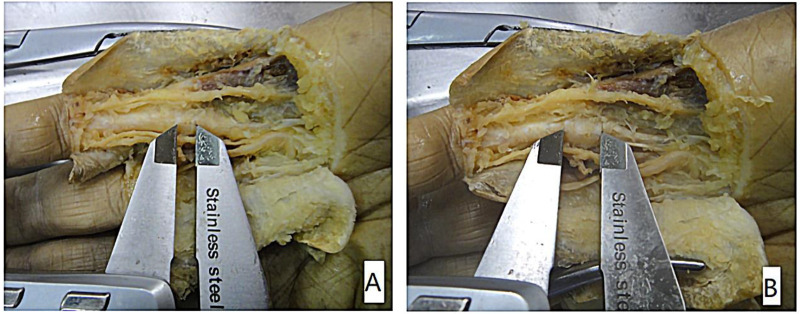
(**A**) the length of the released A1 pulley; (**B**) the actual lengths of the A1 pulley.

There are three kinds of release results: missed release, partial release and full release. The length of the released A1 pulley and actual lengths of the A1 pulley were measured (Figure 5). The full release rate of the A1 pulley (%) = The number of cases of full releases of the A1 pulley ÷ total number of cases ×  100%, The percentage of released A1 pulley was then calculated as percentage of released A1 pulley (%) = length of the released A1 pulley ÷ actual length of the A1 pulleys × 100%. There would be missed releases, The other soft tissues may be released, such as A2 pulley, or PA pulley. If the A1 pulley has a missed release, the length of the release A1 pulley is recorded as 0 mm for data analysis. In group T, due to the lack of continuity of the release, the release of A1 pulley maybe constituted with several cut, so the length of the release A1 pulley is recorded as the sum of several cut lengths.

#### 2.2.3. Data analysis

SPSS software (version 20.0) was used for data analysis. The anatomical measurement data are expressed as mean ± standard deviation (x ± s), and if it conforms to normal distribution and the variance is homogeneous, one-way ANOVA is used; otherwise, nonparametric Kruskal Wallis test is used. The counting data are expressed in (number of cases/percentage), using C2 test or Fisher's precision probability test, and *post hoc* test is used for pairwise comparison between multiple groups. The inspection level is *a* = 0.05.

## 3. Results

### 3.1. Safety

Group U: There were 29 (20.7%) cases of injury. Of these, 25 (17.9%) had a tendon injury. Only four (2.9%) cases had a partial tendon laceration, and the rest had minimal longitudinal tendon scoring without any interruption along the tendon length. Tendon continuity was maintained in all ﬁngers. Two (1.4%) cases had A2 and PA pulley injuries. Two (1.4%) cases had a digital nerve injury. There were no injuries to the vessels (Table 1).

**Table 1:** The injury cases and injury rate of three groups.

	Injury of the flexor digital tendon (cases/percentage)	Injury of the A2 and PA pulley (cases/percentage)	Injury of the digital nerve (cases/percentage)	Injury of the digital vessel (cases/percentage)	Total
Group U (*n* = 140)	25/17.9% (0.66)	2/1.4% (−1.93)	2/1.4% (0.00)	0/0 (−1.0)	29/20.7%
Group N (*n* = 140)	23/16.4% (0.09)	11/7.9% (2.80)	1/0.7% (−0.87)	1/0.7% (0.5)	36/25.7%
Group T (*n* = 140)	20/14.3% (−0.7)	4/2.9% (−0.9)	3/2.1% (0.9)	1/0.7% (0.5)	28/20%
*χ* ^2^	10.790				
*P* value	0.183				
Cramer's V	0.113*				

*P > 0.05. Adjusted residuals appear in parentheses below observed frequencies.

Group N: There were 36 (25.7%) cases of injury. Of these, 23 (16.4%) had a tendon injury. Only one (0.7%) case had a partial tendon laceration, and the rest had minimal longitudinal tendon scoring without any interruption along the tendon length. Tendon continuity was maintained in all ﬁngers. Eleven (7.9%) cases had an A2 and PA pulley injury. One (0.7%) case had a digital nerve injury. One (0.7%) case had vessel injury.

Group T: There were 28 (20.0%) cases of injury. Of these, 20 (14.3%) had a tendon injury. only one (0.7%) had a partial tendon laceration, and the rest had minimal longitudinal tendon scoring without any interruption along the tendon length. Tendon continuity was maintained in all ﬁngers. Four (2.9%) cases had an A2 and PA pulley injury. Three (2.1%) cases had a digital nerve injury. One (0.7%) case had vessel injury.

The total injury rate was highest in the order of group N, group U, and group T, however, differences between groups were not statistically significant.

### 3.2. Efficacy

The released length, actual length of A1 pulley and the percentage of released A1 pulley of three groups showed by Table 2. The data distribution does not conform to normality, so non-parametric test is adopted.

**Table 2:** The release length, actual length of A1 pulley and the percentage of released A1 pulley of three groups.

	L1: The release length of A1 pulley (x ± s, mm)	L2: The actual length of A1 pulley (x ± s, mm)	The percentage of released A1 pulley (L1/L2)
Group U (*n* = 140)	5.7 ± 2.5	7.8 ± 3.1	71.4% ± 30.7%
Group N (*n* = 140)	5.2 ± 1.6	8.1 ± 1.9	66.0% ± 20.3%
Group T (*n* = 140)	4.7 ± 2.5	7.7 ± 2.7	61.0% ± 30.4%
*χ* ^2^	23.220	8.496	12.965
*P*–value	0.000	0.014	0.002
Adj. Sig			
U—T	0.000	1.000	0.002
U—N	0.298	0.025	0.026
T—N	0.006	0.051	1.000

The released lengths of the A1 pulleys(L1) was highest in the order of group U, group N, and group T, There was statistical difference between the three groups. The *post hoc* pairwise comparison using Bonferroni method to correct the significance level found that there was significant difference between group U and group T (adj. *p* = 0.025), group N and group T (adj. *p* = 0.006).

The actual lengths of the A1 pulleys(L2) was highest in the order of group N, group U, and group T, but this was not statistically significant. The comparison between two groups shows that there was significant difference between group U and group N (adj. *p* = 0.025), and no statistically significant between other groups.

The percentage of released A1 pulley of the three groups were compared: group U > group N > group T. There was statistical difference between the three groups. The comparison between two groups shows that there was significant difference between group U and group T (adj. *p* = 0.002), group U and group N (adj. *p* = 0.026), and there is no significant difference between group T and group N.

The missed release rate was highest in the order of group T, group U, and group N, but this was not statistically significant (Table 3). The full release rate was highest in the order of group U, group N, group T. The comparison result: *χ*^2^ = 19.917, *p* = 0.001; There were significant difference in the partial release and full release of A1 pulley between group U and group T, group N.

**Table 3:** The missed release cases and full release rate of three groups.

	Missed release (cases/percentage)	Partial release (cases/percentage)	Full release (cases/percentage)	total
Group U (*n* = 140)	4/2.9%	92/65.7%^a,b^	44/31.4%^a^	140
Group N (*n* = 140)	8/5.7%	110/78.6%	22/15.7%	140
Group T (*n* = 140)	13/9.3%	108/77.1%	19/13.6%	140
Total	25/6.0%	310/73.8%	85/20.2%	420

^a^ The difference between group u and group n is statistically significant.

^b^ The difference between group u and group t was statistically significant.

## 4. Discussion

Lorthioir first described percutaneous A1 pulley release in 1958 in 52 patients (4). The patients reported good results, with no long-term complications. Currently, percutaneous A1 pulley using a needle knife has been widely used in treating TF, with a one-time success rate of 91–98.2% (18–20). However, because of the nonstandard operation and individual ability of the operator, there are still potential safety hazards and cases of treatment failure reported clinically, such as postoperative infection, postoperative haematoma formation, numbness or hypoesthesia after a digital nerve injury, digital tendon injuries, adhesions, and even complete laceration. The main reasons are as follows: lack of direct visualisation or image guidance technology since the surgeon can only determine the position of the needle knife in the tissue by the sensation under the hand, and the A1 pulley is closely adjacent to the flexor digital tendon and neurovascular bundles. If the surgeon is not familiar with the anatomical structure or the needle knife technique, it is easy to potentially injure important structures, including the flexor digital tendon and neurovascular bundles.

Ultrasound-guided percutaneous A1 pulley precise release using a needle knife has received increasing attention in clinical treatment. A good result of ultrasound-guided percutaneous A1 pulley release using a needle knife for TF was reported in two studies published in 2019. Baojian et al. (21) reported a retrospective study of 60 patients with TF from the outpatient department treated using a needle knife under ultrasound guidance. The thickness of the A1 pulley, Quinnell grade, and visual analogue scale scores were collected and analysed preoperatively, 2 weeks after the operation, and 1 month after the operation. The results of the thickness of the A1 pulley, Quinnell grade, and visual analogue scale scores were significantly decreased (*P* < 0.05) compared with those before the operation. Guo Lanqin (22) reported a controlled trial of ultrasound-guided percutaneous A1 pulley release by needle knife and simple drug injection for the treatment of 76 patients with TF. The clinical efficacious rate of ultrasound-guided percutaneous A1 pulley release in the needle knife group was significantly higher than that of the drug injection alone group (85.29% > 61.76%, *P* < 0.05). Ultrasound guidance can effectively reduce the rate of adverse events. However, there are no reports on ultrasound-guided anatomical studies, controlled trials of ultrasound-guided needle knife release, non-ultrasound-guided needle knife release, and classic needle knife release.

In this controlled trial, ultrasound-guided needle knife release, non-ultrasound-guided needle knife release, and classic needle knife release were compared using anatomical methods. The results showed that there was no significant difference in the injury rates among the three groups (*P* > 0.05). Of those injury cases, few cases had a neurovascular injury, and most of those injury cases had minimal longitudinal tendon scoring. This result showed that the three methods of percutaneous A1 pulley release by needle knife are highly safe. However, ultrasound guidance can accurately locate the location of the A1 pulley and accurately identify tendons and blood vessels. It is easier to follow and master for beginners.

The results of the lengths of the released A1 pulleys and the percentage of released A1 pulleys show that ultrasound guidance can significantly improve the accuracy of percutaneous A1 pulley release using a needle knife. The advantages of ultrasound guidance are as follows: before the operation, ultrasound guidance can accurately locate the location of the A1 pulley and accurately identify tendons and blood vessels. During the operation, ultrasound guidance can guide the process of percutaneous A1 pulley release using a needle knife in real time to improve accuracy and security. After the operation, an ultrasound can evaluate the condition of the A1 pulley.

The full release rate was highest in the order of group U, group N, group T. The full release rate is very low even if it is guided by ultrasound (13.6%–31.4%). But in clinic treatment, all clinical triggering might be relieved with release of the thickened A1 pulley. Meanwhile, the release was technically easier with the patients' pulleys than with the cadaveric specimens. A patient's thickened A1 pulley is easier to identify and release and has a more obvious grating sensation when pushing the needle knife. At the same time, whether the release is successful can be judged according to the disappearance of the patient's active triggering at the time of surgery. A total of 20–30% length of the A1 pulley (1.5–2.5 mm) of all cadavers is residual unreleased. In patients with the incomplete release of the A1 pulley, all clinical triggering might be relieved, or symptoms might be ameliorated but perhaps not abolished, as suggested by some clinical studies. Whether the remaining intact A1 pulley would have caused future dysfunction cannot be determined.

There are very few A1 pulley missed released in the three groups. The major reason is that the cadaver specimens were fixed with formalin, cadavers might have altered landmarks and tissue turgor owing to soft tissue shrinkage or ﬂuid shifts postmortem which might affects the imaging quality of the ultrasound, and the operator's judgment of A1 pulley position and the feeling of his hands when releasing. Another reason may be the operator's wrong judgment, and the error in the position selection of the needle-knife entry point will also lead to missed releasing.

The major limitation of the present study is that it was a cadaveric series, which makes the extrapolation of clinical results challenging. Cadavers might have altered landmarks and tissue turgor owing to soft tissue shrinkage or ﬂuid shifts postmortem which directly affects the imaging quality of the ultrasound. The presence of a nodule over the A1 pulley, a thickened pulley, or a history of triggering was not a requisite inclusion in this study. Meanwhile, it is difficult to measure the percentage of released A1 pulley in a live patientfurther studies are required to determine the relationship between the percentage of released A1 pulley of A1 pulley release and the relief of clinical symptoms.

The data from this study suggest that the length and percentage of released A1 pulley is longer by ultrasound-guided percutaneous A1 pulley release using a needle-knife, and there was no statistical difference in the injury rate between the three techniques. We believe that ultrasound-guided percutaneous A1 pulley release using a needle knife is both a safer and more effective method in patients with TF who have had unsuccessful conservative treatment, including steroid injections. Although a minimal longitudinal tendon scoring of the flexor digital tendon can occur; however, this mild degree of injury seems unavoidable and does not affect the function of the patient's fingers; hence, it should not affect the results of the release. At the same time, ultrasound guidance is easier to learn and promote while lowering the psychological burden on patients. We suggest that ultrasound-guided percutaneous A1 pulley release using a needle knife is very efficient for stenosing flexor tenosynovitis and is worthy of clinical popularisation.

## Data Availability

The raw data supporting the conclusions of this article will be made available by the authors, without undue reservation.
